# Predictors of clinical trial data sharing: exploratory analysis of a cross-sectional survey

**DOI:** 10.1186/1745-6215-15-384

**Published:** 2014-10-02

**Authors:** Vinay K Rathi, Kelly M Strait, Cary P Gross, Iain Hrynaszkiewicz, Steven Joffe, Harlan M Krumholz, Kristina Dzara, Joseph S Ross

**Affiliations:** Yale University School of Medicine, 333 Cedar Street, New Haven, CT 06510 USA; Center for Outcomes Research and Evaluation, Yale-New Haven Hospital, 1 Church Street, Suite 200, New Haven, CT 06510 USA; Section of General Internal Medicine and the Robert Wood Johnson Foundation Clinical Scholars Program, Department of Medicine, Yale University School of Medicine, P.O. Box 208093, New Haven, CT 06520 USA; Nature Publishing Group, The Macmillan Building, 4 Crinan St, London, N1 9XW UK; Department of Medical Ethics and Health Policy, University of Pennsylvania Perelman School of Medicine, 3401 Market Street, Suite 320, Philadelphia, PA 19104 USA; Section of Cardiovascular Medicine and the Robert Wood Johnson Foundation Clinical Scholars Program, Department of Medicine, Yale University School of Medicine, P.O. Box 208088, New Haven, CT 06520 USA; Department of Health Policy and Management, Yale University School of Public Health, P.O. Box 208034, New Haven, CT 06520 USA

**Keywords:** Data sharing, Clinical trial, Data repository

## Abstract

**Background:**

A number of research funders, biomedical journals, pharmaceutical companies, and regulatory agencies have adopted policies advocating or mandating that clinical trialists share data with external investigators. We therefore sought to determine whether certain characteristics of trialists or their trials are associated with more unfavorable perceptions of data sharing. To date, no prior research has addressed this issue.

**Methods:**

We conducted an exploratory analysis of responses to a cross-sectional, web-based survey. The survey sample consisted of trialists who were corresponding authors of clinical trials published in 2010 or 2011 in one of six general medical journals with the highest impact factors in 2011. The following key characteristics were examined: trialists’ academic productivity and geographic location, trial funding source and size, and the journal in which it was published. Main outcome measures included: support for data sharing in principle, concerns with data sharing through repositories, and reasons for granting or denying requests. Chi-squared tests and Fisher’s exact tests were used to assess statistical significance.

**Results:**

Of 683 potential respondents, 317 completed the survey (response rate 46%). Both support for data sharing and reporting of specific concerns with sharing data through repositories exceeded 75%, but neither differed by trialist or trial characteristics. However, there were some significant differences in explicit reasons to share or withhold data. Respondents located in Western Europe more frequently indicated they have or would share data in order to receive academic benefits or recognition when compared with respondents located in the United States or Canada (58 versus 31%). In addition, respondents who were the most academically productive less frequently indicated they have or would withhold data in order to protect research subjects when compared with less academically productive respondents (24 versus 40%), as did respondents who received industry funding when compared with those who had not (24 versus 43%).

**Conclusions:**

Respondents indicated strong support for data sharing overall. There were few notable differences in how trialists viewed the benefits and risks of data sharing when categorized by trialists’ academic productivity and geographic location, trial funding source and size, and the journal in which it was published.

**Electronic supplementary material:**

The online version of this article (doi:10.1186/1745-6215-15-384) contains supplementary material, which is available to authorized users.

## Background

A number of major clinical research funders [1–3] and biomedical journals [4–7] have adopted policies supporting or mandating clinical trial data sharing in an effort to maximize the reproducibility and reliability of the results of clinical trials and maximize return on investment in research. Sharing data can increase its value in a number of ways, including by allowing external investigators to investigate secondary hypotheses, aiding future trial design, and providing training resources for students and fellows [8]. Clinical researchers most commonly share data either by depositing it in repositories, with terms of access defined by maintaining organizations, or by granting personal requests for the data on their own terms. Secondary users of trial data, such as the Cochrane Collaboration, are now advocating for stronger data sharing policies as a means to safeguard against selective reporting of outcomes and improve the medical evidence base for clinical decision-making [9–17]. These growing calls for clinical trial data sharing have prompted the European Medicines Agency [18] and several companies in the pharmaceutical industry [19–21] to commit to far-reaching data access policies for the first time. It now appears that many policymakers are no longer contemplating the need to share data, but rather the best way to do so [22]. Indeed, the United States Food and Drug Administration has recently proposed to share de-identified clinical trial data for the first time, albeit on a limited basis and such that product identity would not be disclosed, only therapeutic class [23].

Recently, we conducted a survey of clinical trialists to inform these ongoing data sharing efforts. Despite previous research finding clinical researchers to be amongst the least likely to share data [24], we found strong support for clinical trial data sharing, both in principle and in practice [25]. Most, but not all, respondents to our survey agreed that the clinical research community should promote and facilitate data sharing, and the majority of respondents who were required or requested to share data had done so. However, respondents identified a number of practical concerns with sharing trial data. Because trialists represent a diverse community of investigators with respect to funding sources, research aims and scope, and academic aspirations, they may vary in their concerns or support for data sharing. For instance, trialists receiving industry funding and investigators who do not publish frequently may both be less inclined to share data, albeit for different reasons. Trialists receiving industry funding may withhold data because of restrictions on their control over the data [26–29] or potential conflicts with their research funders, whereas investigators who do not publish frequently may withhold data in an effort to optimize their own academic productivity. Similarly, trialists conducting either very large or small clinical studies may be less inclined to share data for different reasons, as trialists conducting bigger studies may be concerned with receiving adequate return on investment from large-scale undertakings in data collection, and trialists conducting smaller studies may be concerned with protecting patient confidentiality. On the other hand, trialists who published their clinical studies in journals advocating for data sharing and those located in Western Europe may be more inclined to share data in accordance with journal data sharing policies [5, 30], and a cultural shift towards data sharing in their scientific community [18], respectively. To date, no research has addressed this issue.

Accordingly, using the data collected during the previously described survey, we conducted a secondary analysis to explore whether concerns and support for data sharing varied among trialists according to professional background and aspects of the trials they conducted. In particular, we examined the following key characteristics, which we anticipated may influence trialists’ perceptions of data sharing: trialists’ academic productivity, geographic location, and trial characteristics including funding source, size, and the journal in which it was published. We expect these findings will help inform policy-makers by identifying groups of trialists who may be more resistant to new requirements and regulations on data sharing, or who may have specific concerns about data sharing that could be addressed, in order to facilitate data sharing.

## Methods

### Study sample and design

The study sample, design, and data have been reported in detail [25]. In brief, we assembled a sample of clinical trialists (n = 683) using an Ovid Medline search to identify the first corresponding author named in each clinical trial published in 2010 or 2011 in one of six general medical journals with the highest impact factors in 2011 (Journal Citation Reports, Thomson Reuters; *New England Journal of Medicine*, *Lancet*, *Journal of the American Medical Association*, *Annals of Internal Medicine*, *PLoS Medicine*, and *The BMJ*). We also obtained from the article the following information from the original article: trialists’ geographic location and affiliation, trial funding source and size, and the journal in which the study was published. This information was used to compare survey respondents and non-respondents. After soliciting participation from our study sample via email and telephone, a total of 317 corresponding authorscompleted an anonymous web-based survey, yielding a 46% response rate. Participation was voluntary and included an opportunity to win one of five $100 gift certificates from Amazon.com. Approval from the Yale University School of Medicine Human Research Protection Program was obtained before conducting the study (HIC Protocol# 1207010491), and consent was considered to be implied when participants completed the online survey.

### Survey instrument development and domains

The development and domains of the survey instrument have been fully described [25]. Briefly, the design of our 38-item survey instrument (Additional file 1) was informed by a review of the literature [8, 31–39] and discussions among the authors and with experts. The survey was conducted between July and September of 2012 using the Qualtrics™ (Provo, Utah, United States) online platform. The survey instrument assessed support for and prevalence of data sharing, concerns with and reasons for data sharing, and respondent characteristics. Respondents were first asked whether they were required by their research funder to deposit data from their published study in a repository and if they had done so. Next, respondents were asked whether they supported requirements for data sharing in principle, through data repositories, and in response to personal requests. Respondents were then asked about concerns with sharing data through repositories, experiences with receiving and making personal data sharing requests, reasons for granting or declining personal requests, and their beliefs on the right of first use of trial data. Finally, the following self-reported sociodemographic and professional information was obtained: age, sex, primary employer, academic rank (if applicable), geographic location, academic productivity, and funding status.

### Main outcome measures

We examined four main outcome measures to explore differences in support for and concerns about data sharing among clinical trialists. First, we examined support for data sharing in principle, ascertained through the statement ‘The clinical research community should promote and facilitate clinical trial data sharing’; responses were categorized as ‘Agree’ or ’Disagree’. Second, we examined general concerns with sharing data through repositories, ascertained through one of the following three questions, depending upon respondents’ indicated experience with sharing their study data through a repository: ‘If you had been required to share the deidentified data from this study through a data repository, would you have any of the following concerns?’, ‘Since sharing the deidentified data from this study through a data repository, have you had any of the following concerns?’, or ‘In anticipation of sharing the deidentified data from this study through a data repository, do you have any of the following concerns?’. Responses were categorized as ‘Any Concern’ or ‘No Concerns’ and those with concerns were then prompted to select any of the following items as applicable: concerns related to investigator or funder interests, the protection of research subjects, appropriate data use, or other concerns.

Third, we examined general reasons for sharing data in response to personal requests, ascertained through one of the following two questions, depending on respondents’ indicated experience with sharing their study data in response to personal requests: ‘For what general reason(s) did you share the study data?’ or ‘For what general reason(s) would you be most likely to share the study data?’. Those with reasons for sharing data were then prompted to select any of the following items as applicable: administrative requirements, promotion of open science, academic benefits or recognition, or other reasons. Lastly, we examined general reasons for withholding data in response to personal requests. This was ascertained through one of the following two questions, depending on respondents’ indicated experience with withholding their study data in response to personal requests: ‘For what general reason(s) did you not share the study data?’ or ‘For what general reason(s) would you be most likely to not share the study data?’. Those with reasons for withholding data were then prompted to select any of the following items as applicable: protect investigator or funder interests, protect research subjects, ensure appropriate data use, or other reasons.

### Main independent variables

We explored five key characteristics of trialists or their trials in an effort to differentiate support for and concerns about data sharing among clinical trialists: trialists’ academic productivity and geographic location, trial funding source and size, and the journal in which it was published. These characteristics were selected based on a review of the literature, which, among other reasons, identified trialists’ motivation to publish [8, 33, 35], the protection of proprietary interests [32], ineffective journal-mandated policies [35, 40], and concern for patient confidentiality [32] as potential barriers to clinical trial data sharing. These characteristics were identified prior to conducting our exploratory analysis.

Trialists’ academic productivity was assessed by asking respondents how many research articles they had published over the past three years and what percent overall job effort they had devoted to research activities during the 2011 to 2012 fiscal year. Our findings were consistent regardless of how academic productivity was defined, and so results are only presented when defining academic productivity by the number of research articles published over the last three years (≤10 articles, 11 to 25 articles, or >25 articles). Trialists’ geographic location was categorized as United States or Canada, Western Europe, or elsewhere. Trial funding source was categorized as solely funded by government sources, solely funded by for-profit industry sources or mixed funding (which includes industry funding), or solely funded by non-profit sources (such as charitable foundations, professional associations, and universities). Trial size was divided by quartile; of these, the middle two groups were then combined (≤239 study subjects, 240 to 2,016 study subjects, and ≥2,017 study subjects). Lastly, the journal in which the trial was published was assigned as one of the following: *New England Journal of Medicine*, *Lancet*, *Journal of the American Medical Association*, *Annals of Internal Medicine*, *PLoS Medicine*, and *The BMJ*. Among these journals, *Annals of Internal Medicine* [5], *The BMJ* [30], and *PLoS Medicine* [41] have advocated for data sharing, and the *New England Journal of Medicine* has recently begun requiring authors to post clinical trial protocols [42].

### Statistical analysis

The analyses were not pre-specified at the time of survey administration. Rather, they were conducted with the purpose of generating hypotheses for future research efforts seeking to inform the development of data sharing policies. As described previously [25], we used chi-square tests for categorical variables (author employer and geographic location, trial funder, and journal) and the Kruskal-Wallis test for continuous variables (trial enrollment) to compare respondents and non-respondents. We used descriptive statistics to summarize our main outcome measures overall, and by the key characteristics of trialists and their trials described above. Chi-squared tests and Fisher’s exact tests were used to assess statistical significance. In order to account for multiple comparisons of the survey responses for the five main independent variables of interest, we applied the Bonferroni correction, specifying a *P*-value threshold of 0.01 or less to be considered a statistically significant difference. Survey data were analyzed by KMS using SAS version 9.3 (SAS Institute Inc., Cary, North Carolina, United States).

## Results

There were 317 corresponding authors of clinical trials who responded to our survey. The majority of respondents were between 50 and 64-years-old (50%) and male (77%, Table 1). A minority of respondents published 10 articles or fewer over the past three years (22%), with roughly equal numbers publishing between 11 and 25 articles (37%), and more than 25 articles (41%) during the same period. More than half of our respondents were located in United States or Canada (53%), with over one third located in Western Europe (36%), and the remaining located elsewhere (11%). Nearly half of all respondents received industry funding for their study (48%), whereas a much smaller group was supported solely by non-profit funding sources (14%). With respect to trial size, a quarter of respondents were corresponding authors of trials with approximately 240 subjects or fewer, and another quarter with approximately 2,000 subjects or more. The majority of respondents published their trial in *New England Journal of Medicine* (36%), *Lancet* (22%), or *Journal of the American Medical Association* (14%). Approximately one fourth of respondents (28%) published their trial in one of the three journals advocating for data sharing, which included *Annals of Internal Medicine* (16%), *The BMJ* (8%), and *PLoS Medicine* (4%). Survey respondents did not differ from non-respondents with respect to corresponding author location or affiliation, trial enrollment, or journal in which the article was published. However, trial funders did differ between respondents and non-respondents; corresponding authors of trials funded solely by government sources responded more often than corresponding authors of trials funded solely by industry or by mixed funding sources (data not shown, provided in prior publication [25]).

**Table 1:** **Respondent and trial characteristics**

	Respondents (%)
**Age**	
≤49 years	126 (40)
50 to 64 years	159 (50)
≥ 65 years	31 (10)
**Sex**	
Male	243 (77)
Female	73 (23)
**Trialist academic productivity (research articles published in past three years)**	
1 to 10 articles	71 (22)
11 to 25 articles	117 (37)
>25 articles	129 (41)
**Trialist geographic location**	
United States or Canada	167 (53)
Western Europe	113 (36)
Other	37 (11)
**Trial funding source**	
Government	120 (38)
Industry or mixed funding	152 (48)
Other	45 (14)
**Trial size**	
≤239 subjects	80 (25)
240 to 2,016 subjects	158 (50)
≥2,017 subjects	79 (25)
**Journal in which trial was published**	
*NEJM*	113 (36)
*Lancet*	70 (22)
*JAMA*	43 (14)
*Annals of Internal Medicine*	24 (8)
*The BMJ*	53 (16)
*PLoS Medicine*	14 (4)

*JAMA, Journal of the American Medical Association; NEJM, New England Journal of Medicine.*

### Support for data sharing

We found no significant differences in support for data sharing in principle between respondents categorized by trialists’ academic productivity and geographic location, trial funding source and size, and the journal in which it was published (Table 2), as rates of support ranged between 81 and 100%.

**Table 2:** **Respondents’ support of clinical trial data sharing, stratified by trialist and trial characteristics**

	‘The clinical research community should promote and facilitate clinical trial data sharing’			
	Respondents	Agree (%)	Disagree (%)	***P***value
**Overall**	317	278 (88)	39 (12)	
**Trialist academic productivity (number of research articles published in past three years)**				0.22
1 to 10 articles	71	66 (93)	5 (7)	
11 to 25 articles	117	103 (88)	14 (12)	
>25 articles	129	109 (85)	20 (16)	
**Trialist geographic location**				0.17*
United States or Canada	167	141 (84)	26 (16)	
Western Europe	113	104 (92)	9 (8)	
Other	37	33 (89)	4 (11)	
**Trial funding source**				0.07*
Government	120	108 (90)	12 (10)	
Industry or mixed funding	152	127 (84)	25 (16)	
Other	45	43 (96)	2 (4)	
**Trial size**				0.71
≤239 subjects	80	69 (86)	11 (14)	
240 to 2,016 subjects	158	141 (89)	17 (11)	
≥2,017 subjects	79	68 (86)	11 (14)	
**Journal in which the trial was published**				0.01*
*NEJM*	113	92 (81)	21 (19)	
*Lancet*	70	61 (87)	9 (13)	
*JAMA*	43	36 (84)	7 (16)	
*Annals of Internal Medicine*	24	23 (96)	1 (4)	
*The BMJ*	53	52 (98)	1 (2)	
*PLoS Medicine*	14	14 (100)	0 (0)	

*Fisher exact test. *JAMA, Journal of the American Medical Association; NEJM, New England Journal of Medicine.*

### Concerns with sharing data through repositories

Overall, the majority of respondents (76%) reported at least one experiential or hypothetical concern with sharing data from their published study through repositories (Table 3). However, we found no significant differences in overall concern about sharing data through repositories between respondents categorized by trialists’ academic productivity and geographic location, trial funding source and size, and the journal in which it was published, as rates of overall concern ranged between 67 and 84%. In regards to specific concerns with sharing data through repositories, most respondents (65%) identified appropriate data use as a concern, noting issues such as the prevention of misleading secondary analyses and misinterpretation of data. As with overall concern, we found no significant differences in specific concerns with sharing data through repositories between respondents categorized by trialist and trial characteristics.

**Table 3:** **Respondents’ concerns about sharing clinical trial data through a data repository, stratified by trialist and trial characteristics**

	Concerns about sharing clinical trial data through a data repository										
	Respondents	No concern (%)	Any concern (%)	***P***value	Concerns related to investigator or funder interests (%)	***P***value	Concerns related to the protection of research subjects (%)	***P***value	Concerns related to appropriate data use (%)	***P***value	Other concerns (%)
**Overall**	317	77 (24)	240 (76)		129 (41)		91 (29)		205 (65)		35 (11)
**Trialist academic productivity (number of research articles published in past three years)**				0.32		0.09		0.59		0.18	
1 to 10 articles	71	15 (21)	56 (79)		21 (30)		22 (31)		47 (66)		7 (10)
11 to 25 articles	117	25 (21)	92 (79)		52 (44)		36 (31)		82 (70)		10 (9)
>25 articles	129	37 (29)	92 (71)		56 (43)		33 (26)		76 (59)		18 (14)
**Trialist geographic location**				0.88		0.93		0.65		0.70	
United States or Canada	167	41 (25)	126 (75)		68 (41)		45 (27)		111 (66)		21 (13)
Western Europe	113	26 (23)	87 (77)		45 (40)		36 (32)		72 (64)		11 (10)
Other	37	10 (27)	27 (73)		16 (43)		10 (27)		22 (59)		3 (8)
**Trial funding source**				0.18		0.01		0.34		0.52	
Government	120	35 (29)	85 (71)		39 (33)		32 (27)		75 (63)		16 (13)
Industry or mixed funding	152	30 (20)	122 (80)		75 (49)		42 (28)		103 (68)		13 (9)
Other	45	12 (27)	33 (73)		15 (33)		17 (38)		27 (60)		6 (13)
**Trial size**				0.35		0.10		0.40		0.84	
≤239 subjects	80	24 (30)	56 (70)		26 (33)		24 (30)		52 (65)		9 (11)
240 to 2,016 subjects	158	34 (22)	124 (78)		64 (41)		49 (31)		104 (66)		16 (10)
≥2,017 subjects	79	19 (24)	60 (76)		39 (49)		18 (23)		49 (62)		10 (13)
**Journal in which the trial was published**				0.29*		0.57		0.81*		0.31	
*NEJM*	113	26 (23)	87 (77)		48 (42)		30 (27)		79 (70)		13 (12)
*Lancet*	70	11 (16)	59 (84)		31 (44)		18 (26)		48 (69)		5 (7)
*JAMA*	43	14 (33)	29 (67)		16 (37)		15 (35)		26 (60)		5 (12)
*Annals of Internal Medicine*	24	7 (29)	17 (71)		8 (33)		8 (33)		14 (58)		4 (17)
*The BMJ*	53	16 (30)	37 (70)		18 (34)		17 (32)		32 (60)		8 (15)
*PLoS Medicine*	14	3 (21)	11 (79)		8 (57)		3 (21)		6 (43)		0 (0)

*Fisher exact test. *JAMA, Journal of the American Medical Association; NEJM, New England Journal of Medicine.*

### Reasons for sharing data

The majority of respondents (78%) identified the promotion of open science as an experiential or hypothetical reason for sharing data from their published study (Table 4). We found no significant differences in reasons for sharing data between respondents categorized by trialists’ academic productivity and geographic location, trial funding source and size, and the journal in which it was published, with one exception. When respondents were asked if they have or would share data from their published study in order to receive academic benefits or recognition, their responses differed significantly based on geographic location (*P* <0.001). Respondents located in Western Europe responded affirmatively most frequently (58%), as compared to respondents located in the United States or Canada (31%), and elsewhere (43%).

**Table 4:** Respondents’ reasons for sharing clinical trial data*

	Reasons for sharing clinical trial data*							
	Respondents	Administrative requirements (%)	***P***value	Promote open science (%)	***P***value	Academic benefits or recognition (%)	***P***value	Other reasons (%)
**Overall**	317	55 (17)		248 (78)		133 (42)		56 (18)
**Trialist academic productivity (number of research articles published in past three years)**			0.64		0.26		0.94	
1 to 10 articles	71	10 (14)		58 (82)		31 (44)		8 (11)
11 to 25 articles	117	20 (17)		95 (81)		49 (42)		21 (18)
>25 articles	129	25 (19)		95 (74)		53 (41)		27 (21)
**Trialist geographic location**			0.02		0.46		<0.0001	
United States or Canada	167	32 (19)		135 (81)		52 (31)		35 (21)
Western Europe	113	12 (11)		86 (76)		65 (58)		18 (16)
Other	37	11 (30)		27 (73)		16 (43)		3 (8)
**Trial funding source**			0.62		0.26		0.28	
Government	120	24 (20)		99 (83)		45 (38)		17 (14)
Industry or mixed funding	152	24 (16)		113 (74)		65 (43)		28 (18)
Other	45	7 (16)		36 (80)		23 (51)		11 (24)
**Trial size**			0.36		0.82		0.48	
≤239 subjects	80	18 (23)		64 (80)		33 (41)		14 (18)
240 to 2,016 subjects	158	24 (15)		124 (78)		71 (45)		22 (14)
≥2,017 subjects	19	13 (16)		60 (76)		29 (37)		20 (25)
**Journal in which the trial was published**			0.27**		0.55		0.46	
*NEJM*	113	17 (15)		87 (77)		46 (41)		22 (19)
*Lancet*	70	15 (21)		51 (73)		33 (47)		14 (20)
*JAMA*	43	6 (14)		33 (77)		14 (33)		8 (19)
*Annals of Internal Medicine*	24	8 (33)		20 (83)		13 (54)		2 (8)
*The BMJ*	53	8 (15)		46 (87)		20 (38)		7 (13)
*PLoS Medicine*	14	1 (7)		11 (79)		7 (50)		3 (21)

*If respondent had not been asked to share data they were asked to share their hypothetical reasons for sharing. **Fisher exact test. *JAMA, Journal of the American Medical Association; NEJM, New England Journal of Medicine.*

### Reasons for withholding data

The majority of respondents (74%) identified ensuring appropriate data use as an experiential or hypothetical reason for withholding data from their published study (Table 5), noting specific concerns such as mistrust of the data requester’s intent, data not appropriate for the requested purpose, and the potential for misinterpretation and misleading secondary analyses. We found no significant differences in reasons for withholding data between respondents categorized by trialists’ academic productivity and geographic location, trial funding source and size, and the journal in which it was published, with three exceptions. When respondents were asked if they have or would withhold data from their published study in order to protect research subjects, their responses differed significantly based on trialist academic productivity (*P* = 0.01), trial funding source (*P* = 0.003), and journal of trial publication (*P* <0.001). Respondents who were most academically productive (>25 articles published over the past three years) responded affirmatively least frequently (24%), as compared to respondents who published 1 to 10 articles (41%), and 11 to 25 articles (40%). Respondents who received industry funding also responded affirmatively least frequently (24%), as compared to respondents who received government funding (42%), and non-profit funding (44%). In contrast, authors of trials published in *Annals of Internal Medicine* (67%) and *The BMJ* (47%) responded affirmatively more frequently when compared with respondents who published in *New England Journal of Medicine* (27%), *Lancet* (26%), *Journal of the American Medical Association* (35%), and *PLoS Medicine* (21%).

**Table 5:** Respondents’ reasons for withholding clinical trial data*

	Reasons for withholding clinical trial data*							
	Respondents	Protect investigator or funder interests (%)	***P***value	Protect research subjects (%)	***P***value	Ensure appropriate data use (%)	***P***value	Other reasons (%)
**Overall**	317	121 (38)		107 (34)		233 (74)		37 (12)
**Trialist academic productivity (number of research articles published in past three years)**			0.32		0.01		0.31	
1 to 10 articles	71	22 (31)		29 (41)		55 (77)		3 (4)
11 to 25 articles	117	45 (38)		47 (40)		89 (76)		18 (15)
>25 articles	129	54 (42)		31 (24)		89 (69)		16 (12)
**Trialist geographic location**			0.72		0.79		0.40	
United States or Canada	167	66 (40)		57 (34)		123 (74)		22 (13)
Western Europe	113	43 (38)		36 (32)		86 (76)		14 (12)
Other	37	12 (32)		14 (38)		24 (65)		1 (3)
**Trial funding source**			0.65		0.003		0.92	
Government	120	43 (36)		50 (42)		89 (74)		13 (11)
Industry or mixed funding	152	62 (41)		37 (24)		112 (74)		17 (11)
Other	45	16 (36)		20 (44)		32 (71)		7 (16)
**Trial size**			0.45		0.08		0.75	
≤239 subjects	80	26 (33)		35 (44)		58 (73)		7 (9)
240 to 2,016 subjects	158	62 (39)		50 (32)		119 (75)		20 (13)
≥2,017 subjects	79	33 (42)		22 (28)		56 (71)		10 (13)
**Journal in which the trial was published**			0.11		0.0008**		0.86	
*NEJM*	113	50 (44)		30 (27)		85 (75)		15 (13)
*Lancet*	70	22 (31)		18 (26)		49 (70)		10 (14)
*JAMA*	43	11 (26)		15 (35)		34 (79)		4 (9)
*Annals of Internal Medicine*	24	11 (46)		16 (67)		17 (71)		3 (13)
*The BMJ*	53	19 (36)		25 (47)		37 (70)		5 (9)
*PLoS Medicine*	14	8 (57)		3 (21)		11 (79)		0 (0)

*If respondent had not been asked to share data they were asked to share their hypothetical reasons for not sharing. **Fisher exact test. *JAMA, Journal of the American Medical Association; NEJM, New England Journal of Medicine.*

## Discussion

In our survey assessing clinical trialists’ views on data sharing (the first study of this scope) we conducted exploratory analyses to determine whether certain characteristics of investigators and their trials were associated with more favorable perceptions of sharing. No prior study has examined how perceptions of data sharing may differ among individuals in this diverse community of clinical trial investigators. We found few notable differences in how respondents viewed the benefits and risks of data sharing. When respondents were categorized by trialists’ academic productivity and geographic location, trial funding source and size, and the journal in which it was published, respondents consistently indicated strong support for data sharing. Our findings suggest that, among authors of trials published in general medical journals with high impact factors, no particular group of trialists is more likely than others to be resistant to new requirements and regulations on data sharing.

However, while trialists were generally in agreement about the benefits of data sharing, we found several specific differences in their perceptions of data sharing that merit further discussion. With respect to academic productivity, respondents who published most often (>25 articles over the past three years) cited the protection of research subjects as a reason for withholding data less often. Investigators achieving such a high level of academic productivity are likely to have substantial experience handling data, and this finding may reflect their comfort and familiarity with implementing measures to ensure data de-identification. Future data sharing initiatives may be optimized for less experienced trialists by offering educational materials on best practice for data de-identification [31], and making ethical consultation available when there is no explicit consent for data sharing [43].

Respondents who conducted studies funded by industry cited the protection of research subjects as a reason for withholding data less often. These respondents were more often concerned with protecting investigator or funder interests when sharing data through a repository, a finding which lay just inside the region of statistical significance. These findings may in part reflect respondents’ lack of perceived ownership of data or access to data from industry-funded trials [26–29]. Furthermore, these findings suggest that avoiding potential conflicts with corporate research sponsors is of primary importance to industry-funded trialists. Future data sharing policies may address this concern and secure engagement from trialists involved in industry-funded studies by ensuring robust data access, ownership and stewardship agreements and, where applicable, copyright or other licensing agreements [44].

There were also interesting patterns in the reasons respondents noted for sharing data. More than half of respondents located in Western Europe cited the potential for academic benefits or recognition as a reason for sharing data, whereas respondents located in the United States or Canada cited this reason less often. This finding suggests that the professional desirability of data sharing depends on cultural norms within scientific communities, and the community of trialists based in Western Europe has already begun to foster a culture in which investigators recognize that their academic aspirations may be furthered by sharing data. In the near future, data sharing policies which ensure co-authorship rights to data originators [8] may help incentivize more trialists to share data and align their professional interests with those of the public good. However, academic institutions and promotions committees will need to begin rewarding trialists for creating data and sharing it with other investigators if the global culture of academic medicine is to shift from ‘publish or perish’ to more open science [45, 46]. Some funding agencies, including the National Science Foundation, have already begun to consider data as a research product when evaluating investigators [47].

### Limitations

There are several limitations to consider when interpreting our study. First, our analyses were exploratory and conducted *post-hoc* (after the main survey results were analyzed), and were not powered to detect differences between groups of respondents. Second, with respect to the overall survey there are several important considerations concerning the generalizability of our results including social desirability response bias [48, 49], a sample comprised entirely of trialists published in the general medical journals with the highest impact factors, and an overall response rate which was lower than that of some web-based surveys of clinical trial investigators [27, 29, 50], although higher than other such studies [51]. Furthermore, we did not explicitly define ’data’ in our survey, and respondents may thus have interpreted this concept to mean the level of data that they would be most comfortable sharing (for example, data sufficient to remake published tables versus all participant level data). Because of these issues, our findings may overestimate support for and willingness to share data in the clinical trial community. Finally, our study was also limited by the scope of our survey, as we omitted important questions about whether funders have explicitly prohibited data sharing, experiences negotiating data ownership with others, sharing in the context of respondents’ other trials, and the sharing of other study materials (such as intervention manuals, analysis scripts, and output files) [52] in order to reduce response burden.

## Conclusions

In our survey of clinical trialists’ perceptions and experiences with data sharing, we found few notable differences among subgroups of trialists in how they viewed the benefits and risks of data sharing. When respondents were categorized by trialists’ academic productivity and geographic location, trial funding source and size, and the journal in which it was published, strong support for data sharing remained. These findings suggest that no particular group of trialists is more likely to be resistant to new requirements and regulations on data sharing, as the clinical trials research community appears to have adopted a more favorable outlook on sharing. However, support for data sharing among trialists in principle may not translate to practice, as research funders and other stakeholders often figure prominently in the decision of whether or not to share. A better understanding of the context in which trialists conduct research may thus be helpful in identifying and addressing specific, practical barriers to sharing in order to align public and private interests and fully realize the promise of more open science.

## Electronic supplementary material

Additional file 1: **Survey distributed to potential respondents.** (DOCX 29 KB)

## Abbreviations

*NEJM*: *New England Journal of Medicine*
*JAMA*: *Journal of the American Medical Association*
SAS: Statistical Analysis System.
