# Supplementary material for: Predictors of clinical trial data sharing: exploratory analysis of a cross-sectional survey
Source: Trials. 2014 Oct 2;15:384. doi: 10.1186/1745-6215-15-384 (PMC4192345; doi:10.1186/1745-6215-15-384)
Supplement: Supplementary file 1 — Additional file 1: Survey distributed to potential respondents. (DOCX 29 KB) [file 13063_2013_2249_MOESM1_ESM.docx]

**Appendix**: Survey distributed to potential respondents.

For purposes of this study, clinical trial data sharing is defined as any instance in which an investigator makes trial data (published or unpublished) available to an individual with whom he/she is not collaborating. This can be accomplished either indirectly through a data repository or directly in response to a personal request. The data released are assumed to include socio-demographic and clinical information at the patient-level and contain sufficient detail to conduct statistical analyses of the outcome variables.

Please select the most appropriate answer to each of the following questions.

1. The clinical research community should promote and facilitate clinical trial data sharing.

□ Strongly agree □ Somewhat agree □ Somewhat disagree □ Strongly disagree

1. Please briefly explain your answer.

The following questions focus on your perceptions of sharing clinical trial data through data repositories, archives of clinical trial data accessible to external investigators and managed by at least one research funder. For instance, you may be familiar with the data repository managed by the National Heart, Lung, and Blood Institute (NHLBI) of the U.S. National Institutes of Health (NIH).

1. Were you required by the research funder to share data from your study “[insert publication title]” through a data repository? [BIFURCATION QUESTION: If participants answer “Yes” they will be presented with Question #5; If participants answer “No” they will be presented with Question #4.]

□ Yes □ No

1. If you had been required to share the deidentified data from this study through a data repository, would you have any of the following concerns? (Please check all that apply) [NOTE: Question #4 only asked of participants who answered “No” to Question #3.]

□ Concerns Related to Investigator or Funder Interests

□ Concerns Related to the Protection of Research Subjects

□ Concerns Related to Appropriate Data Use

□ Other Concerns

□ No Concerns

4a. Which of the following issues related to investigator or funder interests would be concerning to you and how significant would they be? [TAILORED QUESTION: Participants will only be presented with Question #4a if they selected the corresponding response in root Question #4]

- - Ensuring my ability to publish original research using the data

□ Major concerns □ Minor concerns □ No concerns

- - Ensuring my colleague’s ability to publish original research using the data

□ Major concerns □ Minor concerns □ No concerns

- - Ensuring that I receive sufficient academic/scientific recognition for sharing my clinical trial data

□ Major concerns □ Minor concerns □ No concerns

- - Incurring expenses associated with sharing data (i.e., direct costs)

□ Major concerns □ Minor concerns □ No concerns

- - Spending undue time or effort to prepare data for sharing (i.e., indirect costs)

□ Major concerns □ Minor concerns □ No concerns Protecting commercially sensitive information

□ Major concerns □ Minor concerns □ No concerns

(Optional) Please briefly describe any other issues related to investigator or funder interests that would be concerning to you.

4b. Which of the following issues related to the protection of research subjects would be concerning to you and how significant would they be? [TAILORED QUESTION: Participants will only be presented with Question #4b if they selected the corresponding response in root Question #4]

- - Maintaining patient confidentiality

□ Major concerns □ Minor concerns □ No concerns - Obtaining research subject consent

□ Major concerns □ Minor concerns □ No concerns

(Optional) Please briefly describe any other issues related to the protection of research subjects that would be concerning to you.

4c. Which of the following issues related to appropriate data use would be concerning to you and how significant would they be? [TAILORED QUESTION: Participants will only be presented with Question #4c if they selected the corresponding response in root Question #4]

- - Ensuring clarity of data elements for other investigators

□ Major concerns □ Minor concerns □ No concerns - Ensuring appropriate data use by other investigators

□ Major concerns □ Minor concerns □ No concerns - Preventing misinterpretation or misleading secondary analyses

□ Major concerns □ Minor concerns □ No concerns

(Optional) Please briefly describe any other issues related to appropriate data use that would be concerning to you.

4d. Please briefly describe any other concerns you would have if you had been required to share the deidentified data from this study through a data repository. [TAILORED QUESTION: Participants will only be presented with Question #4d if they selected the corresponding response in root Question #4]

1. Has the data been deposited in a repository? [[NOTE: Question #5 only asked of participants who answered

“Yes” to Question #3; BIFURCATION QUESTION: If participants answer “Yes” they will be presented with Question #6; If participants answer “No” they will be presented with Question #7.]

□ Yes □ No

1. Since sharing the deidentified data from this study through a data repository, have you had any of the following concerns? (Please check all that apply) [NOTE: Question #6 only asked of participants who answered “Yes” to Question #5.]

□ Concerns Related to Investigator or Funder Interests

□ Concerns Related to the Protection of Research Subjects

□ Concerns Related to Appropriate Data Use

□ Other Concerns

□ No Concerns

6a. Which of the following issues related to investigator or funder interests have caused you concern and how significant have they been? [TAILORED QUESTION: Participants will only be presented with Question #6a if they selected the corresponding response in root Question #6]

- - Ensuring my ability to publish original research using the data

□ Major concerns □ Minor concerns □ No concerns

- - Ensuring my colleague’s ability to publish original research using the data

□ Major concerns □ Minor concerns □ No concerns

- - Ensuring that I receive sufficient academic/scientific recognition for sharing my clinical trial data

□ Major concerns □ Minor concerns □ No concerns

- - Incurring expenses associated with sharing data (i.e., direct costs)

□ Major concerns □ Minor concerns □ No concerns

- - Spending undue time or effort to prepare data for sharing (i.e., indirect costs)

□ Major concerns □ Minor concerns □ No concerns - Protecting commercially sensitive information

□ Major concerns □ Minor concerns □ No concerns

(Optional) Please briefly describe any other issues related to investigator or funder interests that have caused you concern.

6b. Which of the following issues related to the protection of research subjects have caused you concern and how significant have they been? [TAILORED QUESTION: Participants will only be presented with Question #6b if they selected the corresponding response in root Question #6]

- - Maintaining patient confidentiality

□ Major concerns □ Minor concerns □ No concerns - Obtaining research subject consent

□ Major concerns □ Minor concerns □ No concerns

(Optional) Please briefly describe any other issues related to the protection of research subjects that have caused you concern.

6c. Which of the following issues related to appropriate data use have caused you concern and how significant have they been? [TAILORED QUESTION: Participants will only be presented with Question #6c if they selected the corresponding response in root Question #6]

- - Ensuring clarity of data elements for other investigators

□ Major concerns □ Minor concerns □ No concerns - Ensuring appropriate data use by other investigators

□ Major concerns □ Minor concerns □ No concerns - Preventing misinterpretation or misleading secondary analyses

□ Major concerns □ Minor concerns □ No concerns

(Optional) Please briefly describe any other issues related to appropriate data use that have caused you concern.

6d. Please briefly describe any other concerns you have had since sharing the deidentified data from this study through a data repository. [TAILORED QUESTION: Participants will only be presented with Question #6d if they selected the corresponding response in root Question #6]

1. In anticipation of sharing the deidentified data from this study through a data repository, do you have any of the following concerns? (Please check all that apply) [NOTE: Question #7 only asked of participants who answered “No” to Question #5.]

□ Concerns Related to Investigator or Funder Interests

□ Concerns Related to the Protection of Research Subjects

□ Concerns Related to Appropriate Data Use

□ Other Concerns

□ No Concerns

7a. Which of the following issues related to investigator or funder interests are concerning to you and how significant are they? [TAILORED QUESTION: Participants will only be presented with Question #7a if they selected the corresponding response in root Question #7]

- - Ensuring my ability to publish original research using the data

□ Major concerns □ Minor concerns □ No concerns

- - Ensuring my colleague’s ability to publish original research using the data

□ Major concerns □ Minor concerns □ No concerns

- - Ensuring that I receive sufficient academic/scientific recognition for sharing my clinical trial data

□ Major concerns □ Minor concerns □ No concerns

- - Incurring expenses associated with sharing data (i.e., direct costs)

□ Major concerns □ Minor concerns □ No concerns

- - Spending undue time or effort to prepare data for sharing (i.e., indirect costs)

□ Major concerns □ Minor concerns □ No concerns - Protecting commercially sensitive information

□ Major concerns □ Minor concerns □ No concerns

(Optional) Please briefly describe any other issues related to investigator or funder interests that are concerning to you.

7b. Which of the following issues related to the protection of research subjects are concerning to you and how significant are they? [TAILORED QUESTION: Participants will only be presented with Question #7b if they selected the corresponding response in root Question #7]

- - Maintaining patient confidentiality

□ Major concerns □ Minor concerns □ No concerns - Obtaining research subject consent

□ Major concerns □ Minor concerns □ No concerns

(Optional) Please briefly describe any other issues related to the protection of research subjects that are concerning to you.

7c. Which of the following issues related to appropriate data use are concerning to you and how significant are they? [TAILORED QUESTION: Participants will only be presented with Question #7c if they selected the corresponding response in root Question #7]

- - Ensuring clarity of data elements for other investigators

□ Major concerns □ Minor concerns □ No concerns - Ensuring appropriate data use by other investigators

□ Major concerns □ Minor concerns □ No concerns - Preventing misinterpretation or misleading secondary analyses

□ Major concerns □ Minor concerns □ No concerns

(Optional) Please briefly describe any other issues related to appropriate data use that are concerning to you.

7d. Please briefly describe any other concerns you have in anticipation of sharing the deidentified data from this study through a data repository. [TAILORED QUESTION: Participants will only be presented with Question #7d if they selected the corresponding response in root Question #7]

1. If required to share data through a data repository, how long after study completion (i.e., final study data set is ready for analysis) should investigators be entitled to the right of first use of the data?

□ No Right of First Use – the rights to data should be released immediately after study completion

□ One Year □ Two Years □ Three Years □ Four Years □ Five Years or More

□ No Time Limit – the right of first use is entitled until the main findings are accepted for publication

1. Assume that research funders take on the following responsibilities:
   1. Provide for the assembly and maintenance of the repository
   2. Cover all monetary expenses (i.e., direct costs) associated with the repository
   3. Oversee the application process to ensure intended data use is appropriate

All authors of a published clinical study should be required to deposit the deidentified data from the studyin a data repository.

□ Strongly agree □ Somewhat agree □ Somewhat disagree □ Strongly disagree

1. (Optional) Do you have any additional comments related to sharing data through data repositories?

The following questions focus on your own experience with sharing clinical trial data in response to personal requests. A personal data sharing request is any situation in which the author of a published clinical study is directly contacted by an individual requesting access to data relating to that publication.

1. Have you received any personal requests to share patient-level clinical trial research data from your study

“[insert study title]”? [BIFURCATION QUESTION: If participants answer “Yes” they will be presented with Questions #12-15; If participants answer “No” they will be presented with Question #18.]

□ Yes □ No

1. How many personal data sharing requests have you received for this study? [NOTE: Question #12 only asked of participants who answered “Yes” to Question #11.]

□ One □ Two □ Three □ Four or More

1. For what purpose(s) were the data request(s) made? (Please check all that apply) [NOTE: Question #13 only asked of participants who answered “Yes” to Question #11.]

□ Systematic review/meta-analysis □ Cost analysis

□ Subgroup analysis of originally published study □ Novel research question

□ Verification of originally published study □ Academically oriented editorial □ Non-academically oriented editorial □ Other purposes (please specify):

□ Did not specify

1. How many personal data sharing requests have you granted for this study? [NOTE: Question #14 only asked of participants who answered “Yes” to Question #11; BIFURCATION QUESTION: If participants answer any response except “None” they will be presented with Question #16; If participants answer “None” they will be presented with Question #19.]

□ None □ One □ Two □ Three □ Four or More

1. How many personal data sharing requests have you declined/refused for this study? [NOTE: Question #15 only asked of participants who answered “Yes” to Question #11; BIFURCATION QUESTION: If participants answer any response except “None” they will be presented with Question #17; If participants answer “None” they will be presented with Question #20.]

□ None □ One □ Two □ Three □ Four or More

1. For what general reason(s) did you share the study data? (Please check all that apply) [NOTE: Question #16 only asked of participants who answered any response except “None” to Question #14.]

□ Administrative Requirements

□ Promote Open Science

□ Academic Benefits or Recognition

□ Other Reasons

16a. For what specific reason(s) related to administrative requirements did you share the study data? (Please check all that apply) [TAILORED QUESTION: Participants will only be presented with Question #16a if they selected the corresponding response in root Question #16]

□ Comply with journal policy on data sharing

□ Comply with employer/research funder policy on data sharing

(Optional) Please briefly describe any other reasons related to administrative requirements.

16b. For what specific reason(s) related to promoting open science did you share the study data? (Please check all that apply) [TAILORED QUESTION: Participants will only be presented with Question #16b if they selected the corresponding response in root Question #16]

□ Belief in open scientific inquiry

□ Promote new research using existing data

□ Enhance robustness of previously conducted research

□ Avoid redundant clinical trial data collection

□ Facilitate student/fellow opportunities for data analysis

(Optional) Please briefly describe any other reasons related to promoting open science.

16c. For what specific reason(s) related to academic benefits or recognition did you share the study data? (Please check all that apply) [TAILORED QUESTION: Participants will only be presented with Question #16c if they selected the corresponding response in root Question #16]

□ Potential to receive additional academic recognition for sharing data

□ Potential to increase the impact of own research

□ Professional or personal relationship with requester

(Optional) Please briefly describe any other reasons related to academic benefits or recognition.

16d. For what other specific reason(s) did you share the study data? [TAILORED QUESTION: Participants will only be presented with Question #16d if they selected the corresponding response in root Question #16]

17. For what general reason(s) did you NOT share the study data? (Please check all that apply) [NOTE: Question

#17 only asked of participants who answered any response except “None” to Question #15.]

□ Protect Investigator or Funder Interests

□ Protect Research Subjects

□ Ensure Appropriate Data Use

□ Other Reasons

17a. For what specific reason(s) related to protecting investigator or funder interests did you NOT share the study data? (Please check all that apply) [TAILORED QUESTION: Participants will only be presented with Question #17a if they selected the corresponding response in root Question #17]

□ Ensure my ability to publish original research using the data

□ Ensure my colleague’s ability to publish original research using the data

□ Monetary expenses associated with sharing data (i.e., direct costs)

□ Time or effort involved in preparing data for sharing (i.e., indirect costs)

□ Protect commercially sensitive information

□ Lack of academic recognition for sharing clinical trial data

□ Prohibited by formal agreement with trial funder

□ Unsure of employer/research funder policy on data sharing

(Optional) Please briefly describe any other reasons related to protecting investigator or funder interests.

17b. For what specific reason(s) related to protecting research subjects did you NOT share the study data? (Please check all that apply) [TAILORED QUESTION: Participants will only be presented with Question #17b if they selected the corresponding response in root Question #17]

□ Protect patient confidentiality

□ Lack of patient informed consent to share

(Optional) Please briefly describe any other reasons related to protecting research subjects.

17c. For what specific reason(s) related to ensuring appropriate data use did you NOT share the study data? (Please check all that apply) [TAILORED QUESTION: Participants will only be presented with Question #17c if they selected the corresponding response in root Question #17]

□ Did not trust data requester’s intent

□ Data not appropriate for requested purpose

□ Potential for misinterpretation of data

□ Potential for misleading secondary analyses

(Optional) Please briefly describe any other reasons related to ensuring appropriate data use.

17d. For what other specific reason(s) did you NOT share the study data? [TAILORED QUESTION: Participants will only be presented with Question #17d if they selected the corresponding response in root Question #17]

1. If you had received a personal request, would you be willing to share patient-level clinical data from this study?

[NOTE: Question #18 only asked of participants who answered “No” to Question #11.]

□ Yes □ No

1. For what general reason(s) would you be most likely to share the study data? (Please check all that apply)

[NOTE: Question #19 only asked of participants who answered “None” to Question #14.]

□ Administrative Requirements

□ Promote Open Science

□ Academic Benefits or Recognition

□ Other Reasons

□ I would never share data if requested

19a. For what specific reason(s) related to administrative requirements would you be most likely to share the study data? (Please check all that apply) [TAILORED QUESTION: Participants will only be presented with Question #19a if they selected the corresponding response in root Question #19]

□ Comply with journal policy on data sharing

□ Comply with employer/research funder policy on data sharing

(Optional) Please briefly describe any other reasons related to administrative requirements.

19b. For what specific reason(s) related to promoting open science would you be most likely to share the study data? (Please check all that apply) [TAILORED QUESTION: Participants will only be presented with Question #19b if they selected the corresponding response in root Question #19]

□ Belief in open scientific inquiry

□ Promote new research using existing data

□ Enhance robustness of previously conducted research

□ Avoid redundant clinical trial data collection

□ Facilitate student/fellow opportunities for data analysis

(Optional) Please briefly describe any other reasons related to promoting open science.

19c. For what specific reason(s) related to academic benefits or recognition would you be most likely to share the study data? (Please check all that apply) [TAILORED QUESTION: Participants will only be presented with Question

#19c if they selected the corresponding response in root Question #19]

□ Potential to receive additional academic recognition for sharing data

□ Potential to increase the impact of own research

□ Professional or personal relationship with requester

(Optional) Please briefly describe any other reasons related to academic benefits or recognition.

19d. For what other specific reason(s) would you be most likely to share the study data? [TAILORED QUESTION:

Participants will only be presented with Question #19d if they selected the corresponding response in root Question #19]

20. For what general reason(s) would you be most likely to NOT share the study data? (Please check all that apply)

[NOTE: Question #20 only asked of participants who answered “None” to Question #15.]

□ Protect Investigator orFunder Interests

□ Protect Research Subjects

□ Ensure Appropriate Data Use

□ Other Reasons

□ I would always share data if requested

20a. For what specific reason(s) related to protecting investigator or funder interests would you be most likely to NOT share the study data? (Please check all that apply) [TAILORED QUESTION: Participants will only be presented with Question #20a if they selected the corresponding response in root Question #20]

□ Ensure my ability to publish original research using the data

□ Ensure my colleague’s ability to publish original research using the data

□ Monetary expenses associated with sharing data (i.e., direct costs)

□ Time or effort involved in preparing data for sharing (i.e., indirect costs)

□ Protect commercially sensitive information

□ Lack of academic recognition for sharing clinical trial data

□ Prohibited by formal agreement with trial funder

□ Unsure of employer/research funder policy on data sharing

(Optional) Please briefly describe any other reasons related to protecting investigator or funder interests.

20b. For what specific reason(s) related to protecting research subjects would you be most likely to NOT share the study data? (Please check all that apply) [TAILORED QUESTION: Participants will only be presented with Question #20b if they selected the corresponding response in root Question #20]

□ Protect patient confidentiality

□ Lack of patient informed consent to share

(Optional) Please briefly describe any other reasons related to protecting research subjects.

20c. For what specific reason(s) related to ensuring appropriate data use would you be most likely to NOT share the study data? (Please check all that apply) [TAILORED QUESTION: Participants will only be presented with Question #20c if they selected the corresponding response in root Question #20]

□ Did not trust data requester’s intent

□ Potential for misinterpretation of data

□ Potential for misleading secondary analyses

(Optional) Please briefly describe any other reasons related to ensuring appropriate data use.

20d. For what other specific reason(s) would you be most likely to NOT share the study data? [TAILORED QUESTION: Participants will only be presented with Question #20d if they selected the corresponding response in root Question #20]

1. Have you ever made a personal request to another investigator with whom you were not collaborating to share patient-level clinical trial research data from a previously conducted study? [SKIP QUESTION: If participants answer “Yes” they will be presented with Questions #22-25; If participants answer “No” the survey skips to Question #26.]

□ Yes □ No

1. How many personal data sharing requests have you made to other clinical investigators? [NOTE: Question #22 only asked of participants who answered “Yes” to Question #21.]

□ One □ Two □ Three □ Four or More

1. For what purpose(s) did you make the data sharing request(s)? (Please check all that apply) [NOTE: Question #23 only asked of participants who answered “Yes” to Question #21.]

□ Systematic review/meta-analysis □ Cost analysis

□ Subgroup analysis of originally published study □ Novel research question

□ Verification of originally published study □ Academically oriented editorial

□ Nonacademically oriented editorial □ Other purposes (please specify):

1. How many of your personal data sharing requests were granted? [NOTE: Question #24 only asked of participants who answered “Yes” to Question #21.]

□ None □ One □ Two □ Three □ Four or More

1. How many of your personal data sharing requests were declined/refused? [NOTE: Question #25 only asked of participants who answered “Yes” to Question #21.]

□ None □ One □ Two □ Three □ Four or More

1. Assume personal data sharing requests operate under the following conditions:
   1. The data requesters would cover all monetary expenses (i.e., direct costs) associated with sharing data in response to personal requests.
   2. Investigators would be under no obligation to release data in response to a personal request if they do not trust the data requester’s intent or determine that the data may not be used appropriately.

All authors of a published clinical study should be required to share the deidentified data relating to that publication in response to personal requests.

□ Strongly agree □ Somewhat agree □ Somewhat disagree □ Strongly disagree

1. (Optional) Do you have any additional comments related to sharing data in response to personal requests?

The final questions focus on you.

1. Please indicate your age range.

□ 34 years or younger □ 35-49 years □ 50-64 years □ 65 years or older

1. Please indicate your gender.

□ Male □ Female

1. How many years have passed since the completion of your highest professional degree?

□ 0-9 years □ 10-24 years □ 25 years or more

1. Where did you receive scientific training while completing your highest professional degree?

□ United States or Canada □ Western Europe □ Elsewhere

1. Which of the following best classifies your current primary employer? [SKIP QUESTION: If participants answer “Yes” they will be presented with Questions #33; If participants answer “No” the survey skips to Question #34.]

□ Academic Institution □ Private Industry □ Non-Profit Organization

□ For-Profit Hospital □ Government

1. Which of the following best describes your academic rank? [NOTE: Question #33 only asked of participants who answered “Academic Institution” to Question #32.]

□ Lecturer, Fellow, or Student □ Affiliated Professor □ Assistant Professor

□ Associate Professor □ Full Professor

1. What percent of your overall job effort did you devote to research time during fiscal year 2010-2011?

□ Less than 25% □ 25% - 49% □ 50% - 74% □ 75% or greater

1. Over the past three years, approximately how many articles have you published in peer reviewed journals?

□ 1-5 □ 6-10 □ 11-25 □ More than 25

1. Over the past three years, approximately how many research grants and contracts (either internally or externally funded) from any source have you been awarded on which you are the principal investigator? [SKIP QUESTION: If participants answer any response except “None” they will be presented with Questions #37-38; If participants answer “None” the survey ends.]

□ None □ 1-3 □ 4-6 □ 7-10 □ More than 10

1. What type of organizations awarded these research grants and contracts? (Please check all that apply) [NOTE: Question #37 only asked of participants who answered any response except “None” to Question #36.]

Internal funding (funding from your employer):

□ Government □ Non-profit organizations □ Private industry

External funding (funding from sources outside of your employer):

□ Government □ Non-profit organizations □ Private industry

1. Over the past three years, what was the total direct cost associated with these grants and contracts? [NOTE: Question #38 only asked of participants who answered any response except “None” to Question #36.]

□ Less than $50,000 □ $50,000 - $499,999 □ $500,000 - $999,999 □ $1,000,000 - $4,999,999 □ More than $5,000,000
